# Association of physical capacity with heart rate variability based on a short-duration measurement of resting pulse rate in older adults with obesity

**DOI:** 10.1371/journal.pone.0189150

**Published:** 2017-12-21

**Authors:** Chun-De Liao, Jau-Yih Tsauo, Dun-Jen Hsiao, Tsan-Hon Liou, Shih-Wei Huang, Li-Fong Lin

**Affiliations:** 1 School and Graduate Institute of Physical Therapy, College of Medicine, National Taiwan University, Taipei, Taiwan; 2 Department of Physical Medicine and Rehabilitation, Shuang Ho Hospital, Taipei Medical University, New Taipei City, Taiwan; 3 College of Public Health and Nutrition, Taipei Medical University, Taipei, Taiwan; 4 Graduate Institute of Injury Prevention and control, Taipei Medical University, Taipei, Taiwan; 5 Department of Physical Medicine and Rehabilitation, School of Medicine, College of Medicine, Taipei Medical University, Taipei, Taiwan; 6 School of Gerontology Health Management, College of Nursing, Taipei Medical University, Taipei, Taiwan; University of Illinois at Urbana-Champaign, UNITED STATES

## Abstract

**Background:**

Obesity can limit physical capacity and lower physical activity levels in elderly people. Low physical activity levels may be mediated by autonomic dysfunction with decreased heart rate variability (HRV). However, the relationship between autonomic dysfunction and low physical capability remains unclear. This cross-sectional study investigated the association of low physical capability with HRV in older adults with obesity.

**Materials and methods:**

We recruited 231 old man and 210 old women with a mean (range) age of 65.5 (51−78) and 62.9 (52−76) years, respectively. Physical capability was measured using mobility tasks, including functional reach, single-leg stance (SLS), gait speed (GS), timed up and go, and timed chair rise (TCR), and the scores on these tasks were merged and transformed into a global physical capability score (GPCS). HRV was measured using a 7-min resting pulse-based technique, and the time- and frequency-domain indices of HRV were obtained including standard deviation of normal-to-normal intervals (SDNN), root mean square of successive differences at rest (rMSSD), and high-frequency (HF) power. All HRV indices were natural log (ln) transformed for analysis. Participants were divided into high, moderate, and low physical-capability groups according to their physical performance. Multivariate analysis of covariance was performed to test differences in HRV indices among physical-capability groups with participants’ characteristics serving as covariates. A stepwise regression model was established to identify the determinants of HRV indices. We used hierarchical regression analysis to identify the association of the GPCS with HRV indices.

**Results:**

In both men and women, the low physical-capability group exhibited significantly increased heart rate (*P* <0.05) and decreased HRV in terms of a decreased ln[SDNN] (*P* <0.001), ln[rMSSD] (*P* <0.05) and ln[HF] (*P* <0.05), compared with the high physical-capability group. GS positively predicted ln[SDNN], whereas SLS, GS, and TCR were determinants of ln[HF], regardless of gender. The GPCS in older men and women independently accounted for 29.9% (*P* <0.001) and 23.7% (*P* <0.001), respectively, in variance in ln[SDNN].

**Conclusions:**

A low physical-capability level is an independent determinant of decreased HRV in older adults with obesity.

## Introduction

Obesity is a burden on the elderly population, and it contributes to an increased risk of many medical problems, such as diabetes mellitus and cardiometabolic disease, that eventually have a substantial negative impact on an individual’s health status [1–3]. Obesity is characterized by a proinflammatory state initiated by adipocytes, and the adiposity-engendered systemic inflammation is closely linked to metabolic risk factors such as hyperinsulinemia and hyperglycemia [3, 4]. These risk factors further impair body homeostasis through a series of hemodynamic consequences [2, 4]. In addition, geriatric individuals with obesity may have a high risk of muscle loss and a related decline in physical function, with underlying changes in body composition [5–7].

The autonomic nervous system is believed to play a critical role in mediating the pathophysiology of obesity [8–12]. Autonomic dysfunction can be assessed in terms of heart rate variability (HRV), which has become a marker for the risk stratification of cardiovascular consequences because of its clinical relevance to mortality [13, 14]. HRV can be measured by using an alternative assessment tool based on short-duration measurements of pulse rate variability [15, 16]. Deteriorations in HRV are cholinergic and observed in association with abnormal glucose regulation, adiposity-induced immune responses, and low physical domain values for quality of life [17–19], all of which lead to increased allostatic load and adverse health outcomes [20–23].

Although cardiometabolic impairment (a combination of metabolic and cardiovascular dysfunction) is the hallmark of obesity, studies have suggested that physical function should also be considered in order to fully manage obesity in the elderly population [24, 25]. Impaired physical functioning is a critical determinant of disability and mortality in older adults [26–28], and obesity can further exacerbate the age-related decline in physical function. The risk of adverse physical consequences such as self-reported physical difficulty [6, 7] and reduced physical capability [5, 7, 25], a parameter that can be objectively measured to describe a person’s ability to perform free-living activities including walking and rising from a chair [29], is higher in older adults with obesity than in their lean peers. Self-reported physical difficulty or compromised functional capability in older people with obesity can be attributed to reduced cardiorespiratory fitness and impaired muscle function engendered by underlying cardiac autonomic dysfunction and skeletal muscle stress [6, 25, 30–32]. These factors may lead to further physical inactivity [33] and potentially hinder community-dwelling older people from leaving their houses daily [34].

A large body of evidence reveals an association of HRV with numerous physical domain measures including self-rated physical fitness [35], physical activity levels (particularly moderate-to-vigorous levels) [35–40], physical inactivity [39], and self-reported physical functional status [19]. Nevertheless, the association of HRV with objectively measured physical capability in older adults remains unclear. Considering that physical activity levels in older adults closely depend on performance-based physical function [41–44] and that mobility disability is becoming prevalent in older populations with obesity [25], it is important to understand the relationship between HRV and physical capability in elderly people with obesity.

The aim of this study was to determine whether objectively measured physical capability levels have an association with HRV in older adults with obesity. We hypothesized that older adults with higher physical capabilities would have significantly higher HRV than do those with lower physical capabilities, and that physical capability levels would be significantly associated with variations in HRV.

## Materials and methods

### Design

We conducted an observational, cross-sectional study at the Metabolic Management Center of the Physical Medicine and Rehabilitation Department of Shuang Ho Hospital, Taipei Medical University. Potential participants were sequentially recruited from a health screen admission in the community during a time period from June 2015 to February 2016. The eligibility of all recruited patients was determined through a medical chart review and body fat assessment at screening admission. During the examination, all eligible participants underwent subjective evaluation through questionnaires, and their HRV and physical capability levels were measured. All measurements were performed by a trained research assistant. This study was approved by the Joint Institutional Review Board of Taipei Medical University (protocol number: N201602035), and each participant provided written informed consent before the examination.

### Participants

**Fig 1:**
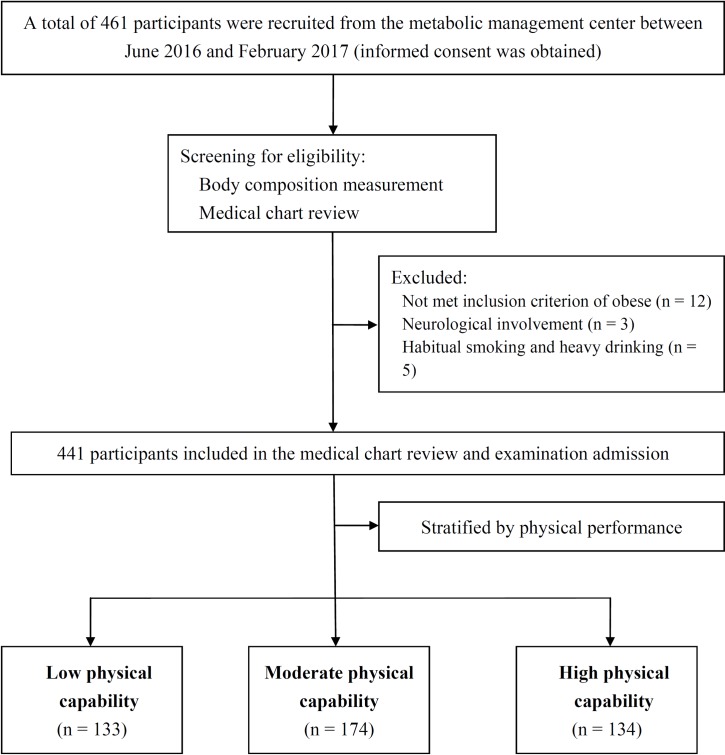
Flowchart depicting participant enrollment.

Participants were selected according to the guidelines of the STROBE statement extension for observational studies [45], as outlined in the flowchart in Fig 1. In this study, obesity was measured using the method of Baumgartner [46]. At screening admission, body fat percentage (BF%), which is considered a valid estimator of body composition, was measured using an eight-polar bioelectrical impedance analysis device with a multifrequency current (Inbody 220, Biospace, Seoul, Republic of Korea) [47]. BF% values of >27% for men and >38% for women were considered the cutoff points for obesity in older populations [48].

The inclusion criteria for participants in this study are outlined as follows: (1) being aged between 50 and 80 years and (2) having a BF% of >27% for men and >38% for women. Participants were excluded if they met the following exclusion criteria: (1) with a BF% not meet the gender specific cut points for obesity; (2) receiving a diagnosis of neurological disorders, orthopedic, or rheumatologic problems that affect the execution of mobility tasks; (3) having history of heart disease, serious arrhythmia, or pacemaker use; (4) habitual smoking or heavy consumption of stimulant beverages such as alcohol; (5) consuming any medication that can presumably influence autonomic functions, such as antihypertensives, anticholinergics, or antidepressants, for at least 1 month prior to the beginning of the study; and (6) having difficulty in lying supine for 10 min.

During the examination, participants’ characteristics, including age and sex, were recorded. Body height, weight, BMI, and BF% were measured using the aforementioned eight-polar bioelectrical impedance analysis device. In addition, the comorbidity status of each participant was evaluated using the Comorbidity Illness Rating Scale (CIRS), which addresses all relevant body systems and was validated for use in older people [49].

### Physical capability assessment

Physical capability was assessed through the measurement of functional mobility tasks including functional reach (FR) [50], single-leg stance (SLS) [51], gait speed (GS) [51], timed up and go (TUG) [52], and timed chair rise (TCR) [53, 54]; these tasks have been used as indicators in older adult populations [55] and validated in such populations [51–54, 56]. Each participant performed a practice trial before the test, and two test trials were conducted. The mean score of the two test trials for each task was subsequently calculated for statistical analysis.

#### Functional reach

FR provides a dynamic measure of balance, regardless of the movement strategy selected [50]. However, previous studies using the FR test have reported that body height is a significant confounding factor for the test results [57, 58]; for example, Isles et al. found that the FR distance increased by 3.3 cm for every 10 cm of body height [58]. Therefore, we used the FR distance–to–participant height ratio (FHR), as described previously by Hageman et al. [57], to minimize the confounding effect engendered by the intragroup variation in body height, according to the following formula: FHR = [FR distance (cm)/height (cm)] × 100.

#### Single-leg stance

SLS assesses balance control, and the SLS score represents the total time for which a person can stand on his or her dominant leg. In this study, SLS tasks were performed by participants with their eyes open.

#### Gait speed

GS (in m/s) measures the time required for people to walk for a predetermined distance. In this study, we measured the time taken by participants to walk for 10 m on a track at a self-determined pace [51].

#### Timed up and go

TUG measures the time required for people to rise from a chair, walk around, and then return to the chair. In this study, TUG was determined as the time taken by participants to rise from their chairs (height, 42 cm; depth, 26 cm), walk for 3 m, turn around, and return to a seated position in the chair at a self-determined speed. A walking aid was used by participants during the test if necessary.

#### Timed chair rise

TCR measures the ability to get out of a chair. In this study, participants stood upright from a seated position in a chair (height, 45 cm), with their arms folded across their chest, and returned to a seated position as many times as possible within a 30-s period.

Each of the five mobility tests (i.e., FHR, SLS, GS, TUG, and TCR) was attributed a score of 1–4 by using quartiles of performance with the fourth, third, second, and first quartiles being coded as 4, 3, 2, and 1 points, respectively). Participants who performed three or more of the five mobility tasks scored as 1 were classified as having low physical capability, whereas those who performed three or more of the five mobility tasks scored as 4 were classified as having high physical capability. The remaining participants were classified as having moderate physical capability.

In the present study, a global physical capability score (GPCS) was adapted from an approach proposed in previous studies [5, 59]. The scores of all five tests were then summed to produce a GPCS for each participant; scores ranged from 5 to 20. One advantage of the GPCS, compared with individual tests, is that it provides an overall measure of a participant’s performance while taking into account several tasks related to daily activities.

### HRV analysis

HRV was measured immediately prior to mobility tests. Participants were first asked to rest quietly for 10 min in a supine position; subsequently, they were assisted with attaching the ANSWatch monitor (Taiwan Scientific Co., Taipei, Taiwan) to their left wrists and were instructed to close their eyes, relax, and remain quiet. A time of approximately 7 min was required in each ANSWatch test to determine the HRV and hemodynamic indices, including blood pressure (BP) and resting heart rate (HR). All data were extracted to a computer by using ANSWatch Manager Pro software.

The ANSWatch wrist monitor, with multiple piezoelectric sensors in the cuff, has been employed to measure BP waveforms in the radial artery [60, 61]. HRV analysis was performed using pulse cycle intervals instead of RR intervals. HRV variables, including time- and frequency-domain variables, were analyzed according to the international standard [62]. The time-domain HRV variables comprised the standard deviation (SD) of normal-to-normal (NN) intervals (SDNN, ms) and the root mean square of successive differences at rest (rMSSD, ms). The frequency-domain HRV variable, namely high-frequency power (HF, 0.15–0.4 Hz, ms^2^), was determined using a power spectral analysis. All HRV indices were natural log (ln) transformed for analysis.

### Sample size estimation

The sample size estimation of this study was based on primary outcomes and SDNN values and was performed using G-Power 3 [63]. At a statistical power of 0.95, an effect size of 0.20 [38], and an alpha value of 0.05, we determined that a minimum of 390 participants were required to identify a difference in the SDNN value of 3 ms^2^ among the three participant groups, assuming an SDNN value SD of 14.4 ms^2^ [38].

### Statistical analysis

All analyses were separately performed by gender. One-way analysis of variance and the chi-squared test were used to compare participants’ characteristics among the three physical capability groups. Multivariate analysis of covariance was also performed to examine between-group differences in HRV indices and all mobility measures, with age, BMI, and the CIRS serving as covariates. The Gaussian distribution was tested for all the variables by using the Kolmogorov–Smirnov test. Natural log (ln) transformation was used if data were not normally distributed.

A Pearson product-moment correlation coefficient (*r*) was used to assess the linear relationship between HRV indices and potential factors. When controlling for participants’ characteristics, a multiple stepwise linear regression of the collapsed data was established for each HRV index to identify the association between the HRV index and all mobility measures; in this analysis, each HRV index was log transformed and treated as the dependent variable and all the mobility measures (i.e., FFR, SLS, GS, TUG, and TCR) were treated as exploratory covariates for each model.

The hierarchical regression models were established to explore the association of resting HR and GPCS with each HRV index, after adjusting for control variables related to HRV [64]. Separate hierarchical models were used for each of the 3 dependent HRV variables (i.e., ln SDNN, ln rMSSD, and ln HF). Each model had 3 steps. In step 1, we entered the covariates (i.e., age, BMI, and CIRS). In step 2, we entered resting HR. In step 3, we entered GPCS. Finally, multicollinearity was tested using tolerance and variance inflation factors. SPSS Statistics for Windows (version 17.0; SPSS Inc., Chicago, IL, USA) was used for all analyses, and comparison results with *P* < 0.05 were considered to represent statistically significant differences.

## Results

A total of 461 participants who were referred from the metabolic management center were assessed for their eligibility after they provided informed consent. After excluding 20 participants who did not meet the inclusion criteria (five men and seven women who have a BMI ranging from 24.3 to 25.7 Kg/m^2^did not match the gender specific cutoff points of BF% for obesity, three had an old onset of stroke, and five were habitual smokers, heavy drinkers, and consumers of coffee), we included 231 old man and 210 old women with a mean (range) age of 65.5 (51−78) and 62.9 (52−76) years, respectively, for further assessment (Fig 1).

Table 1 presents the participants’ demographic characteristics and scores on the mobility tasks, according to their group assignment. Overall, both of male and female participants in the high-physical-capability group were younger and had a lower BMI, BF%, CIRS score, and resting HR (all *P* < 0.05) than did those in the low-physical-capability group. In addition, both of male and female participants in the high-physical-capability group exhibited significantly higher performance in mobility tests compared with those in the low-physical-capability group (all *P* < 0.05). Moreover, female participants exhibited generally poor performance than the male peers in mobility.

**Table 1:** Demographic characteristics of participants stratified on age and gender.

	Men									Women								
Items	Low PC			Moderate PC			High PC			Low PC			Moderate PC			High PC		
	mean	±	SD	mean	±	SD	mean	±	SD	mean	±	SD	mean	±	SD	mean	±	SD
n	68			104			59			65			70			75		
Age (years)	69.9	±	8.1	64.8	±	6.4^a^	61.9	±	7.2^a^	68.9	±	5.3	63.8	±	6.4^a^^b^	61.7	±	5.8^a^^b^
BMI (kg/m^2^)	30.4	±	1.6	28.9	±	1.4^a^	29.1	±	1.7^a^	31.4	±	2.1^b^	29.4	±	1.9^a^	28.5	±	1.2^a^^b^
BF%	37.3	±	5.6	34.7	±	6.4^a^	33.5	±	5.4^a^	39.3	±	6.2	37.6	±	7.1	36.1	±	5.9^a^^b^
CIRS score	10.4	±	4.6	7.8	±	3.9^a^	5.4	±	2.1^a^	13.4	±	7.1^b^	8.5	±	5.1^a^	7.3	±	5.9^a^^b^
BP_SYS (mmHg)	131.8	±	10.1	130.4	±	13.1	128.3	±	11.5	132.3	±	12.1^b^	130.4	±	13.1	128.3	±	11.5^b^
BP_DIA (mmHg)	86.5	±	5.4	86.8	±	8.5	87.1	±	6.9	88.1	±	8.4^b^	85.7	±	8.2	84.5	±	7.9^b^
Resting HR (beats/min)	71.8	±	6.5	69.2	±	8.2^a^	66.8	±	7.5^a^	72.3	±	7.4	69.7	±	8.6^a^	65.4	±	7.3^a^
Age group, n (%)																		
<60 years	12		(17.6)	29		(27.9)	29		(49.2)	11		(16.9)	30		(42.9)	33		(44.5)
60–70 years	28		(41.2)	48		(46.2)	19		(32.2)	30		(46.2)	27		(38.6)	28		(37.3)
>70 years	28		(41.2)	27		(26.0)	11		(18.6)	24		(36.9.7)	13		(18.6)	14		(18.7)
Mobility measures																		
FHR	7.3	±	1.9	14.1	±	3.3^a^	18.8	±	4.1^a^	6.7	±	2.9	12.1	±	3.8^a^^b^	18.0	±	2.4^a^
SLS (s)	9.5	±	2.3	15.2	±	3.6^a^	20.6	±	1.9^a^	8.5	±	1.9^b^	12.2	±	3. ^a^^b^	17.5	±	2.3^a^^b^
GS (m/s)	0.9	±	0.2	1.2	±	0.3^a^	1.5	±	0.3^a^	0.8	±	0.2	1.2	±	0.2^a^^b^	1.4	±	0.3^a^
TUG (s)	11.1	±	1.6	8.3	±	2.2^a^	6.6	±	1.9^a^	11.8	±	1.9	9.0	±	2.1^a^^b^	5.5	±	1.6^a^^b^
TCR (repetition)	7.4	±	1.6	14.2	±	3.7^a^	18.5	±	4.1^a^	6.2	±	1.4^b^	11.9	±	3.4^a^^b^	16.4	±	2.7^a^^b^
GPCS	7.3	±	0.8	13.4	±	2.6^a^	18.8	±	0.7^a^	6.0	±	0.8^b^	11.8	±	2.4^a^^b^	18.4	±	0.5^a^

PC = physical capability; BMI = body mass index; CIRS = Cumulative Illness Rating Scale; BP_SYS = systolic blood pressure; BP_DIA = diastolic blood pressure; HR = heart rate; FHR = ratio of the functional reach distance to body height; SLS = single-leg stance; GS = gait speed; TUG = timed up and go; TCR = timed chair rise; GPCS = global physical capability score.

^a^A significant difference compared with the low-physical-capability group, *P* < 0.05.

^b^A significant difference compared with the corresponding physical-capability group in men, *P* < 0.05.

**Fig 2:**
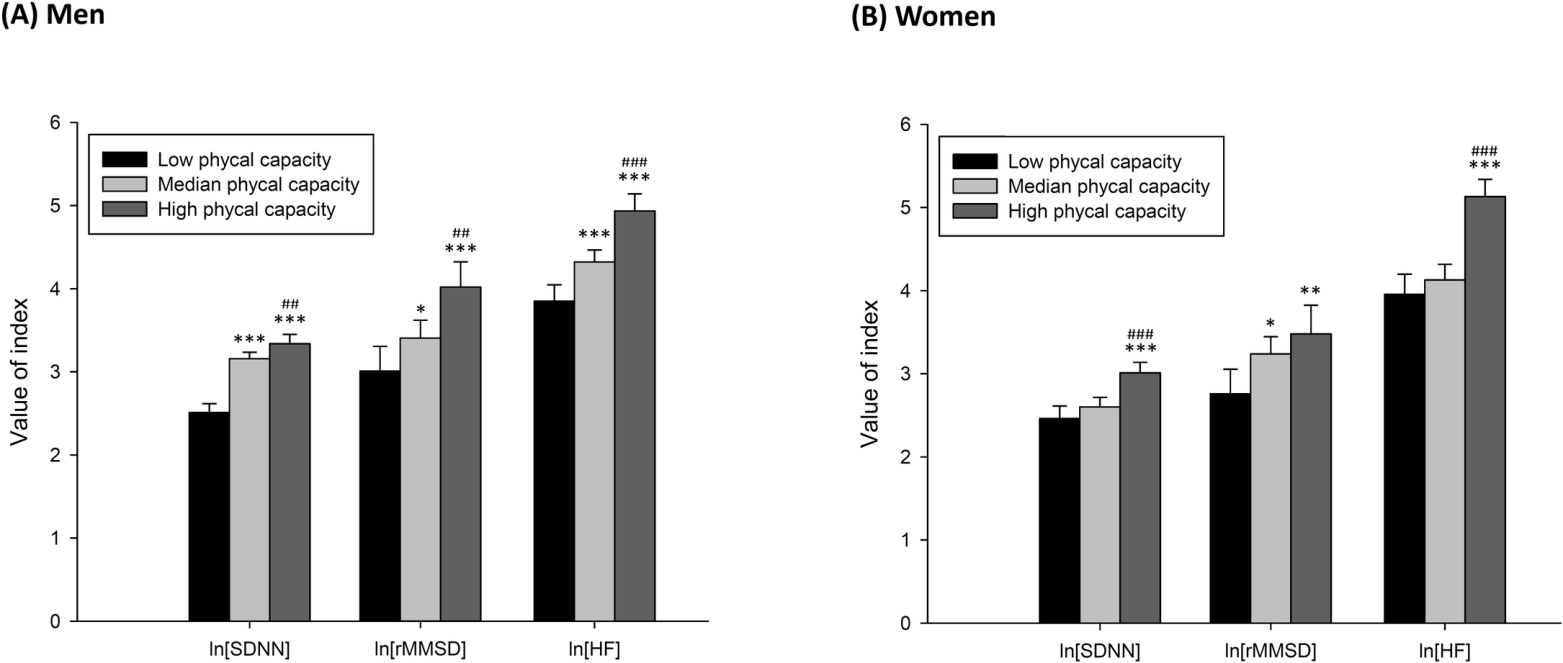
**Adjusted mean values of heart rate variability among the three physical capability groups in old men (A) and women (B).** All HRV indices were natural log transformed. All data were adjusted for age, body mass index, and comorbidity scores. The Y-axis represents the adjusted mean value. The error bar represents the 95% confidence interval. *indicates a significant difference compared with the low physical-capability group, *P* <0.05; ** *P <*0.01; *** *P <*0.001. ^#^indicates a significant difference compared with the moderate-physical-capability group, *P* <0.05; ^##^ *P <*0.01; ^###^ *P <*0.001. SDNN = standard deviation of normal-to-normal (NN) intervals; rMSSD = root mean square of successive differences at rest; HF = high-frequency power.

Primary data of all HRV indices are presentated in S1 Table. Adjusted mean values for the ln transformed HRV indices of the three physical capability groups are showed in Fig 2. The older men with low physical capability exhibited significantly decreased ln SDNN [adjusted mean difference (aMD), −0.83; *P* <0.001], ln rMSSD (aMD, −0.74; *P* = 0.006), and ln HF (aMD, −1.09; *P* <0.001), compared with their high physical-capability peers. Similarly, the older women who had low physical capability showed significantly lower ln SDNN (aMD, −0.55; *P* <0.001), ln rMSSD (aMD, −0.83; *P* = 0.008), and ln HF (aMD, −1.18; *P* <0.001), compared with the high physical-capability group.

Table 2 shows the correlation between the HRV indices and potential moderators. Age, BMI, comorbidity score, and resting HR exhibited significant correlations with all HRV indices with moderate to low correlation coefficients (all *P* < 0.05) in both older men and women. In addition, all mobility scores in older men were significantly correlated with ln SDNN, ln rMSSD, and ln HF (all *P* < 0.05) as well as older women did. The GCPS in older men was moderately correlated with ln SDNN and ln HF by a correlation coefficient of 0.61(*P* < 0.001) and 0.59 (*P* < 0.001), respectively; similar results was observed in older women.

**Table 2:** Pearson correlation of HRV indices with potential factors and physical mobility, as assessed through regression analyses.

Variables^a^	Men			Women		
	ln[SDNN]	ln[rMSSD]	ln[HF]	ln[SDNN]	ln[rMSSD]	ln[HF]
Age	−0.52***	−0.29***	−0.40***	−0.32***	−0.26***	−0.36***
BMI	−0.22**	−0.27***	−0.31***	−0.31***	−0.29***	−0.20**
CIRS score	−0.22**	−0.14*	−0.21**	−0.35**	−0.28***	−0.19**
Resting HR	−0.43***	−0.32***	−0.38***	−0.33***	−0.43***	−0.30***
Physical mobility						
FHR	0.61***	0.28***	0.46***	0.56***	0.45***	0.53***
SLS	0.45***	0.17*	0.51***	0.57***	0.22**	0.59***
GS	0.65***	0.36***	0.53***	0.41***	0.39***	0.51***
TUG	−0.38***	−0.22**	−0.42***	−0.28***	−0.26***	−0.34***
TCR	0.55***	0.26***	0.45***	0.50***	0.14*	0.45***
GPCS	0.61***	0.29***	0.59***	0.53***	0.37***	0.57***

All HRV variables are nature log transformed (ln).

*P < 0.05

** *P <* 0.01

*** *P <* 0.001.

^a^HRV = heart rate variability; BMI = body mass index; CIRS = Cumulative Illness Rating Scale; HR = heart rate; SDNN = standard deviation of normal-to-normal (NN) intervals; rMSSD = root mean square of successive differences at rest; HF = high-frequency power; FHR = ratio of the functional reach distance to body height; SLS = single-leg stance; GS = gait speed; TUG = timed up and go; TCR = timed chair rise; GPCS = global physical capability score.

Table 3 presents the association between the HRV indices and mobility functioning. In older men, increased SLS, GS, and TCR were independent determinants of increased ln SDNN and ln HF, whereas ln rMSSD was positively associated with GS only. In older women, increased ln HF was significantly associated with most of mobility performances, including SLS, GS, TUG, and TCR (all *P* < 0.05).

**Table 3:** Stepwise linear regression analyses for all indices of heart rate variability.

Dependent	Men^b^					Women^b^				
variables^a^	FHR	SLS	GS	TUG	TCR	FHR	SLS	GS	TUG	TCR
ln[SDNN]	0.14	0.45***	0.39***	-	0.67***	0.53***	0.68***	0.34*	-	0.26
ln[rMSSD]	-	-	0.26**	-	-	0.68***	-	-	-	0.51**
ln[HF]	-	0.61***	0.33***	-	0.29*	-	0.93***	0.32**	-0.13*	0.67*

All HRV variables are natural log transformed (ln).

*P < 0.05

** *P <* 0.01

*** *P <* 0.001.

^a^SDNN = standard deviation of normal-to-normal (NN) intervals; rMSSD = root mean square of successive differences at rest; HF = high-frequency power; FHR = ratio of the functional reach distance to body height; SLS = single-leg stance; GS = gait speed; TUG = timed up and go; TCR = timed chair rise.

^b^Stepwise linear regression variables included mobility task measures. The linear model coefficients are represented as standardized coefficient (β) values. Model was adjusted for age, body mass index, and comorbidity scores.

Table 4 shows the association of resting HR and GPCS with all HRV indices. In old men, resting HR explained an additional 12.6% [*F* (1, 226) = 48.73, *P* < 0.001], 3.6%, [*F* (1, 226) = 11.33, *P* < 0.01], and 6.8% [*F* (1, 226) = 21.56, *P* < 0.001] of variation in ln SDNN, ln rMSSD, and ln HF, respectively, as well as old women did. The GPCS in old men independently explained an additional 29.9% [*F* (1, 225) = 232.58, *P* < 0.001], 2.5% [*F* (1, 225) = 7.95, *P* < 0.01], and 10.6% [*F* (1, 225) = 39.43, *P* < 0.001] of the variance of ln SDNN, ln rMSSD, and ln HF, respectively; similar results were observed in old women.

**Table 4:** Hierarchical regression analyses for heart rate variability.

Dependent	Step	Independent	Men					Women				
variables^a^		variables^a^	*R*^2^	Adjusted *R*^2^	*R*^2^ change	*F* change	*df*	*R*^2^	Adjusted *R*^2^	*R*^2^ change	*F* change	*df*
ln[SDNN]	1	Age, BMI, CIRS	0.29	0.28	0.29	30.78***	3, 227	0.43	0.42	0.43	51.54***	3, 206
	2	Resting HR	0.42	0.40	0.13	48.73***	1, 226	0.51	0.50	0.08	33.74***	1, 205
	3	GPCS	0.71	0.71	0.30	232.58***	1, 225	0.75	0.74	0.24	194.54***	1, 204
ln[rMSSD]	1	Age, BMI, CIRS	0.24	0.23	0.24	23.48***	3, 227	0.03	0.02	0.03	2.24	3, 206
	2	Resting HR	0.27	0.26	0.04	11.33**	1, 226	0.09	0.07	0.06	12.61***	1, 205
	3	GPCS	0.30	0.28	0.03	7.95**	1, 225	0.12	0.10	0.04	8.68**	1, 204
ln[HF]	1	Age, BMI, CIRS	0.22	0.21	0.22	21.27***	3, 227	0.14	0.12	0.14	10.94***	3, 206
	2	Resting HR	0.29	0.27	0.07	21.56***	1, 226	0.21	0.20	0.07	19.22***	1, 205
	3	GPCS	0.39	0.38	0.11	39.43***	1, 225	0.35	0.33	0.13	41.73***	1, 204

All HRV variables are natural log transformed (ln).

*P < 0.05

** *P <* 0.01

*** *P <* 0.001.

^a^HRV = heart rate variability; SDNN = standard deviation of the normal-to-normal (NN) intervals; rMSSD = root mean square of successive differences at rest; HF = high frequency power; BMI = body mass index; CIRS = cumulative illness rating scale score; HR = heart rate; GPCS = global physical capability score.

## Discussion

The present study investigated the association between physical capability and 7-min resting pulse-based HRV in older adults with obesity. Our results reveal an association of a lower physical capability level with HRV components, including decreased ln SDNN, ln rMSSD, and ln HF in older men and women with obesity. Consistent with these findings, after controlling for potential confounding factors, we observed that the objective measures of functional mobility (i.e., FHR, GS, SLS, TCR, and TUG) were independent predictors of low HRV, and that physical capability (i.e., GPCS) significantly contributed to the variance in the HRV in both older men and women with obesity.

The accuracy levels of pulse-based HRV and ECG-based HRV measurements were compared. The application of pulse-based HRV measurements for assessing healthy individuals at rest was demonstrated to be adequately accurate, whereas mental stress tended to reduce the agreement between two assessment techniques [16]. The current study applied 7-min resting pulse-based HRV rather than ECG-based HRV, which has been used in most of the previous studies evaluating associations between clinical disorders and autonomic dysfunction. Despite being obese with higher BF% values, all of our participants were relatively healthy, after the exclusion of diagnoses of neurological disorders and heart disease. Because we excluded participants who received medications that might influence autonomic activities at least a month prior to this study, we did not assess participants’ mental stress; this may bias the results of this study. However, each participant was asked to rest in a lying supine position for 10 min before HRV testing.

Previous several studies have indicated an association of physical activity levels with HRV in older people. Specifically, studies regarding short-term HRV analysis and method have reported that a higher physical activity level was associated with increased SDNN [36, 40, 65], rMMSD [37, 40, 65], and HF [36, 40, 65] in older adults; in addition, previous studies have identified a clear relationship between high physical activity and short-term HRV in individuals with overweight and obesity [36, 66]. The findings of our present study also indicate that physical capability, as derived from objectively measured mobility tasks, was significantly associated with HRV measures, regardless of age, sex, or comorbidities. Despite some discussion [67], the mechanisms through which autonomic dysfunction mediates the physical domain of health (particularly physical function) in individuals with obesity remain unclear. However, in older adults with overweight and obesity, reduced physical capability can be linked to the effect of excessive adiposity on muscles, which plays a crucial role in metabolic adaptation in obesity [68–70]. In addition, the autonomic nervous system dysfunction in elderly individuals with obesity was marked by low muscle mass, which further highlights the relationship between autonomic dysfunction and muscle impairment [66]. Lack of measuring muscle mass did not allow us to directly draw the relationships between low muscle mass and low physical capability. However, our participants with older age and higher BF% in the low physical-capability group than their high physical-capability peers may be closely associated with muscle attenuation [71]. Taken all of above together, these findings may explain the associations of diminished HRV with low physical capability and low mobility performance.

Another possible exploration involves the adaptation of physical fitness and whole-body energy expenditure indicated by resting HR [72]. A higher resting HR is predictive of poorer physical fitness, which may also be associated with low HRV in older individuals [73]. Compared with the high-physical-capability group, HRV markedly decreased in the low-physical-capability group, presumably reflecting an adaptation to lower physical fitness in terms of a significantly higher resting HR. In addition, our results showed that both resting HR and GPCS have significant contributions in explaining the variance in HRV. Therefore, our results may indicate that both the state of physical fitness and physical capability are associated with HRV.

### Study limitations

The current study has some limitations that should be considered. First, because of the cross sectional design of this study, we could not identify causality within existing associations. However, the participants’ current physical capability levels could be assessed, and the associations between physical capability levels and pulse-based HRV were significant after adjustment for age and other confounders. Second, the participants selected for this study were a generally healthy group of older adults. Therefore, our findings may not be generalizable to the whole population in this age range, and this should be considered when examining HRV in people with certain illnesses or diseases such as metabolic syndrome or frailty. Third, there are some potential confounders such as lower limb arthritis which limits physical capacity and sleep-disordered breathing which is prevalent in obese older adults and can have a large effect on daytime functioning and probably daytime HRV; in addition, the participants’ mental and cognitive status was not assessed, and no psychological indices were employed in this study; nevertheless, HRV can be simultaneously influenced by various physiological and psychological conditions. Although people who were taking psychological medications were assessed and excluded, subclinical psychological or mental stress can be a potential confounding factor, and the derived associations may thus have then been overestimated. Fourth, we did not have any way to monitor HR and HRV over testing time; therefore, we could not ensure whether participants start to fall asleep, by which a decrease in HR and an increase in HRV can be observed, during the whole testing procedure. Maintaining not to fall asleep during the 10-min pretest rest and the 7-min testing period in a lying supine position may be difficult for older adults. Fifth, all of our mobility tests are related to non-aerobic fitness. It should be accounted that aerobic fitness also has contributions counting for an individual’s physical capacity in daily life. Finally, although we identified several significant associations through a series of regression analyses, the level of correlation appeared to be either moderate or weak, with a regression coefficient of <0.70. The physiological relevance and clinical significance of these associations must be further confirmed.

### Conclusions

The results of this study demonstrate that low physical capability levels identified using objectively measured mobility tasks were associated with low resting pulse-based HRV in older adults with obesity. Given the effect of low HRV on the physical domain of health, we suggest that higher emphasis should be placed on the physical fitness and capability of older individuals with obesity with regard to their activities of daily living. The relevance of this study may be its indication of more close associations between physical capability and autonomic cardiovascular function in older individuals with obesity.

## Supporting information

S1 Table Heart rate variability of participants stratified on age and gender. (DOCX)
