# Correction: Association of physical capacity with heart rate variability based on a short-duration measurement of resting pulse rate in older adults with obesity

**DOI:** 10.1371/journal.pone.0191842

**Published:** 2018-01-19

**Authors:** Chun-De Liao, Jau-Yih Tsauo, Dun-Jen Hsiao, Tsan-Hon Liou, Shih-Wei Huang, Li-Fong Lin

In the Funding section, one of the grant numbers from Taipei Medical University-Shuang Ho Hospital, Ministry of Health and Welfare is missing. The correct grant numbers are 105-SHH-HCP-06 and 106TMU-SHH-05.

There are errors in the author affiliations. The affiliations should appear as shown here: Chun-De Liao^1,2^, Jau-Yih Tsauo^1^, Dun-Jen Hsiao^3^, Tsan-Hon Liou^2,4,5^, Shih-Wei Huang^2,5,6^, Li-Fong Lin^2,7^. **1** School and Graduate Institute of Physical Therapy, College of Medicine, National Taiwan University, Taipei, Taiwan, **2** Department of Physical Medicine and Rehabilitation, Shuang Ho Hospital, Taipei Medical University, New Taipei City, Taiwan, **3** College of Public Health and Nutrition, Taipei Medical University, Taipei, Taiwanm, **4** Graduate Institute of Injury Prevention and control, Taipei Medical University, Taipei, Taiwan, **5** Department of Physical Medicine and Rehabilitation, School of Medicine, College of Medicine, Taipei Medical University, Taipei, Taiwan, **6** Graduate Institute of Sports Science, National Taiwan Sport University, Taoyuan, Taiwan **7** School of Gerontology Health Management, College of Nursing, Taipei Medical University, Taipei, Taiwan.

## References

[1] Liao C-D, Tsauo J-Y, Hsiao D-J, Liou T-H, Huang S-W, Lin L-F (2017) Association of physical capacity with heart rate variability based on a short-duration measurement of resting pulse rate in older adults with obesity. PLoS ONE 12(12): e0189150 https://doi.org/10.1371/journal.pone.0189150 29267296 10.1371/journal.pone.0189150 PMC5739389

