# Supplementary material for: Association of physical capacity with heart rate variability based on a short-duration measurement of resting pulse rate in older adults with obesity
Source: PLoS One. 2017 Dec 21;12(12):e0189150. doi: 10.1371/journal.pone.0189150 (PMC5739389; doi:10.1371/journal.pone.0189150)
Supplement: S1 Table — (DOCX) [file pone.0189150.s001.docx]

Supplementary data

| **Table S1. Heart rate variability of participants stratified on age and gender** | | | | | | | | | | | | | | | | | | | | | | | |
| --- | --- | --- | --- | --- | --- | --- | --- | --- | --- | --- | --- | --- | --- | --- | --- | --- | --- | --- | --- | --- | --- | --- | --- |
|  | Men | | | | | | | | | | |  | Women | | | | | | | | | | |
| Items^a^ | 50–59 years | | |  | 60–69 years | | |  | 70–79 years | | |  | 50–59 years | | |  | 60–69 years | | |  | 70–79 years | | |
|  | mean | ± | SD |  | mean | ± | SD |  | mean | ± | SD |  | mean | ± | SD |  | mean | ± | SD |  | mean | ± | SD |
| n | 80 |  |  |  | 85 |  |  |  | 66 |  |  |  | 98 |  |  |  | 79 |  |  |  | 33 |  |  |
| SDNN, ms | 25.9 | ± | 8.5 |  | 21.7 | ± | 8.9 |  | 16.7 | ± | 10.2 |  | 24.6 | ± | 8.4 |  | 20.5 | ± | 12.4 |  | 14.7 | ± | 9.0 |
| rMMSD, ms | 40.1 | ± | 20.1 |  | 28.5 | ± | 15.1 |  | 22.1 | ± | 15.8 |  | 46.8 | ± | 17.4 |  | 27.1 | ± | 17.5 |  | 21.6 | ± | 16.4 |
| HF, ms^2^ | 172.0 | ± | 87.4 |  | 134.2 | ± | 72.8 |  | 122.2 | ± | 72.7 |  | 175.7 | ± | 67.0 |  | 144.2 | ± | 86.7 |  | 133.3 | ± | 95.2 |
| ^a^SDNN = standard deviation of normal-to-normal (NN) intervals; rMSSD = root mean square of successive differences at rest; HF = high-frequency power. | | | | | | | | | | | | | | | | | | | | | | | |
